# A Novel Putative Role of TNK1 in Atherosclerotic Inflammation Implicating the Tyk2/STAT1 Pathway

**DOI:** 10.1155/2020/6268514

**Published:** 2020-07-10

**Authors:** Mei-Hua Bao, Qiao-Li Lv, Hai-Gang Li, Yi-Wen Zhang, Bao-Feng Xu, Bin-Sheng He

**Affiliations:** ^1^Academician Workstation, Changsha Medical University, Changsha 410219, China; ^2^Science Research Center, Changsha Medical University, Changsha 410219, China; ^3^Department of Science and Education, Jiangxi Key Laboratory of Translational Cancer Research, Jiangxi Cancer Hospital, 519 Beijing East Road, Nanchang, Jiangxi 330029, China; ^4^Department of Pharmacy, Zhejiang Provincial People's Hospital of Hangzhou Medical College, Hangzhou 310010, China; ^5^Department of Neurosurgery, the First Hospital of Jilin University, Changchun 130021, China

## Abstract

**Objective:**

Atherosclerosis is a chronic inflammatory disease which is responsible for many clinical manifestations. The present study was to investigate the anti-inflammatory functions and mechanisms of TNK1 in atherosclerosis.

**Methods:**

The ApoE(-/-) mice and human carotid endarterectomy (CEA) atherosclerotic plaques were used to investigate the differential expression of TNK1. The ApoE(-/-) mice were fed with high-fat diet (HFD) or normal-fat diet (NFD) for 8 weeks; the aorta was separated and stained with oil red O to evaluate the formation of atherosclerosis. TNK1 in mice aorta was measured by qPCR. The human CEA were obtained and identified as ruptured and stable plaques. The level of TNK1 was measured by qPCR and Western-blot staining. Further studies were conducted in THP-1 cells to explore the anti-inflammatory effects of TNK1. We induced the formation of macrophages by incubating THP-1 cells with PMA (phorbol 12-myristate 13-acetate). Afterwards, oxidized low-density lipoprotein (oxLDL) was used to stimulate the inflammation, and the secretion of inflammatory factors was measured by ELISA and qPCR. The levels of TNK1, total STAT1 and Tyk2, and the phosphorylation of STAT1 and Tyk2 were measured by western blot to uncover the mechanisms of TNK1.

**Results:**

The oil red O staining indicated obvious deposition of lipid on the aorta of ApoE(-/-) mice after 8-week HFD treatment. The TNK1 level was much higher in both the HFD-fed ApoE(-/-) mice aorta arch and the ruptured human CEA plaques. We found that TNK1 was highly expressed in THP-1 cells, compared to other atherosclerotic related cells (HUVEC, HBMEC, and HA-VSMC), indicating TNK1 might be involved in the inflammation. Suppressing the expression of TNK1 by shTNK1 inhibited the oxLDL-induced secretion of inflammatory factors, such as IL-12, IL-6, and TNF-*α*. ShTNK1 also inhibited the uptake of lipid and decreased the cellular cholesterol content in THP-1 cells. Furthermore, the shTNK1 suppressed the oxLDL-induced phosphorylation of Tyk2 and STAT1.

**Conclusion:**

TNK1 participated in the inflammation in atherosclerosis. shTNK1 suppressed the oxLDL-induced inflammation and lipid deposition in THP-1 cells. The mechanism might be related to the Tyk2/STAT signal pathway.

## 1. Introduction

Atherosclerosis is a chronic inflammatory disease which is responsible for many clinical manifestations, such as coronary artery disease, and ischemic stroke [1]. During atherogenesis, the monocytes migrate from circulation to intima and change into macrophages. These macrophages secrete a large amount of proinflammatory factors, internalize lipid, and drive the hyperplasia of intima. The inflammation in atherosclerotic lesion leads to the progress and rupture of plaques [2]. The inhibition of the inflammation is an important strategy to prevent the progress of atherosclerosis.

The Janus tyrosine kinase/signal transducers and activators of the transcription (JAK/STAT) pathway is a crucial signal pathway in inflammation [3]. It mediates the production of a large number of cytokines and growth factors. JAK is a nonreceptor tyrosine kinase. In the JAK family, JAK1, JAK2, JAK3, and Tyk2 are the main members. The activation of JAKs stimulates the phosphorylation of STAT proteins. The activated STAT then translocates to the cell nucleus and regulates the transcription of cytokines. The JAK/STAT plays critical roles in atherogenesis [4, 5]. Regulating the JAK/STAT pathway is a potential method for the inhibition of inflammation.

The tyrosine kinase nonreceptor 1 (TNK1) is a novel nonreceptor tyrosine kinase demonstrated to regulate the activation of Tyk2 in JAK/STAT signal. TNK1 belongs to the ACK family. It is expressed in B-lymphomas, hepatocytes, and some cancer cells [6–8]. In response to hepatitis C virus (HCV) infection, the TNK1 is recruited and phosphorylated. The activated TNK1 then phosphorylates the Tyk2/STAT1 pathway. The activated STAT1 regulates the expression of over 300 IFN-stimulated genes (ISGs) and contributes to the controlling of HCV infection [7]. Due to the important roles of STAT1 signal in atherosclerotic inflammation, we hypothesize that TNK1 might participate in the atherogenesis. We also found a significant increase of TNK1 in the aorta of high-fat diet- (HFD-) fed ApoE(-/-) mice (5.78-folds in HFD mice compared to normal fat diet mice) [9]. This inspired us to investigate the functions of TNK1 in the inflammation of atherosclerosis. The present study was to investigate the anti-inflammation effects and mechanisms of TNK1 in atherosclerosis and the involvement of Tyk2/STAT1 signal in the functions of TNK1.

## 2. Methods and Materials

### 2.1. Materials

The human umbilical vein endothelial cells (HUVEC, CRL-1730), human brain microvascular endothelial cells (HBMEC, CRL-3245), human aorta-vascular smooth muscle cells (HA-VSMC, CRL-1999), and human monocytes THP-1 (TIB-202) cell lines were purchased from the American Type Culture Collection (Manassas VA, USA); The oil red O solution was obtained from Beyotime Biotechnology (Shanghai, China); the shRNA lentivirus vector and the polybrene were obtained from the Cyagen Bioscience Inc., (Suzhou, China); IL-12, IL-6, and TNF-*α* ELISA kits were provided by Boster Biological Technology Co. Ltd. (Wuhan, China); Tissue Total cholesterol Assay Kit was from Applygen Technologies Inc. Beijing, China); PrimeScript RT reagent Kit, SYBR Premix DimerEraser™ (Perfect Real Time) assay kit, and the primers were purchased from Takara (Dalian, China); the primary antibodies p-TNK1 (D46E7), TNK1 (C44F9), p-STAT1 (58D6), STAT1 (D1K9Y), p-Tyk2 (Y1054), and Tyk2 (9312S) were purchased from CST (Danvers, MA, USA); the primary antibody for *β*-actin (AP0060) was purchased from Bioworld Technology (Nanjing, China); and the oxLDL was provided by the Peking Union-Biology, Co. Ltd (Beijing, China).

### 2.2. Animals and Diet

The 6-week-old homozygous male ApoE(-/-) mice were obtained from Beijing Vital River Laboratory Animal Technology Co., Ltd. (Beijing, China). The animals were cultivated and treated as previously reported [9]. The body weights of the mice were 22-24 g. The mice were cultivated in a room with the temperature at 22 ± 0.8°C, humidity of 55 ± 10%, and with 12 h light-dark cycles. The experimental procedures were approved by the Ethics Committee of Changsha Medical University, China. The animals were divided randomly into two groups. One group was fed with normal-fat diet (NFD, 4% (*w*/*w*) fat with no cholesterol), another with high-fat diet (HFD, 22.5% (*w*/*w*) fat and 1.25% (*w*/*w*) cholesterol). After 8 weeks of feeding, the mice were anesthetized and perfused with ice-cold normal saline. The aorta was collected, was fixed by 4% paraformaldehyde, and was stained by oil red O solution. The formation of atherosclerotic plaques was observed under an optical microscope. The TNK1 mRNA expression was analyzed by qPCR as described in Section 2.7.

### 2.3. Patients and Samples

21 patients were involved in the present study, including 9 patients with stable plaque and 12 with ruptured plaque. All patients were conducted carotid endarterectomy (CEA) surgery at the First Hospital of Jilin University (Changchun, Jilin, China) from July to November, 2019. The experiments were approved by the Ethics Committee of the First Hospital of Jilin University (No. 2019-272). Written informed consent was obtained from every participant. The stable and ruptured plaques were evaluated by two independent researchers based on the classification presented by American Heart Association (AHA) [10]. All of the surgical specimens were collected and stored in liquid nitrogen until use. The expression of TNK1 mRNA and protein was detected by qPCR and western blot as described in Section 2.7 and Section 2.8.

### 2.4. THP-1 Cell Culture and Transduction

The THP-1 cells were cultivated in RPMI-1640 medium with 10% fetal bovine serum (FBS); HA-VSMC cells were cultivated in F-12K medium with 10% FBS; HBMEC cells were cultivated in DMEM:F12 with 40 *μ*g/mL endothelial growth supplement (ECGS) and 10% FBS; HUVEC cells were in F-12K medium with 0.1 mg/mL heparin, 40 *μ*g/mL ECGS, and 10% FBS. The cells were cultivated in a condition with a humidified atmosphere and 5% CO_2_ at 37°C.

The THP-1 cells were firstly transducted with blank lentivirus vector shScr or TNK1 shRNA lentivirus vector with the MOI of 1 : 10. The polybrene (5 *μ*g/ml) was used to facilitate the transduction. After 24 hours, the medium was replaced with fresh medium to obtain the stable transducted THP-1 sh-TNK1 or shScr cells. The transducted cells were then treated with PMA (20 ng/mL) for 48 hours to form macrophages. The macrophages were treated with 50 *μ*g/mL oxidized low-density lipoprotein (oxLDL) for another 24 hours. The supernatant and cells were collected for further analysis.

### 2.5. Oil Red O Staining and Intracellular Cholesterol Content Measurement

The shScr or shTNK1 transducted cells were washed twice with PBS, fixed by 4% paraformaldehyde for 30 min and stained with oil red O for 15 min. After washing with PBS, the cells were observed under a microscope to evaluate the lipid uptake by the cells [11]. The intracellular total cholesterol was detected by the Tissue Total cholesterol Assay Kit. The cells were collected and then lysed by ultrasonic. The total cholesterol was detected as the instructions indicated.

### 2.6. ELISA Measurement for IL-12, IL-6, and TNF-*α* Secretion

The THP-1 macrophages were transducted with shScr or shTNK1 and then treated with oxLDL as described in Section 2.4. The supernatant was obtained. The IL-12, IL-6, and TNF-*α* in the supernatant were analyzed using the ELISA method according to the manufacturer's instructions.

### 2.7. qPCR Detection for TNK1, Tyk2, STAT1, IL-12, IL-6, and TNF-*α* mRNA Expression

The expressions of TNK1, Tyk2, STAT1, IL-12, IL-6, and TNF-*α* mRNA were detected by qPCR as previously described [12]. Briefly, the total RNA from cells or tissues was extracted using Trizol. Then, the total RNA was reversely transcripted to cDNA using the PrimeScript RT reagent Kit according to the instructions of the manufacturer. The qPCR amplification was conducted on the ABI QuantStudio5 system using the SYBR Premix DimerEraser™ (Perfect Real Time) assay kit. The PCR program was as follows: 95°C for 30 s, followed by 40 cycles of 95°C 5 s, 60°C 30 s. The primers used for the detection are shown in Table 1. The results were analyzed using the 2^(-*ΔΔ*Ct)^ method. *β*-Actin or GAPDH was used as the internal reference.

### 2.8. Western-Blot Analysis of TNK1, P-TNK1, STAT1, P-STAT1, Tyk2, P-Tyk2 Expressions

Total proteins were extracted using RIPA solution supplied with protease/phosphatase inhibitor cocktail. After normalization by the protein concentration, the proteins were separated by SDS-PAGE and transferred to polyvinylidene difluoride (PVDF) membrane. The membranes were then blocked by 5% BSA solution for 1 hour and incubated with primary rabbit monoclonal antibodies for p-TNK1 (D46E7), TNK1 (C44F9), p-STAT1 (58D6), STAT1 (D1K9Y), p-Tyk2 (Y1054), and Tyk2 (9312S), and *β*-actin (AP0060) for overnight in 4°C. After incubation, the membranes were washed with TBS and incubated with second antibody at dilution of 1 : 10000. Then, they were detected by the enhanced chemiluminescence system using the ECL assay kit and analyzed by Quantity One^R^ software.

### 2.9. Statistical Analysis

All the statistics of three independent experiments were presented in the form of mean ± S.D. The significance of the differences was analyzed by ANOVA followed by Newman-Student-Keuls test. A value of *P* < 0.05 is considered statistically significant.

## 3. Results

### 3.1. Expression of TNK1 in HFD-Fed ApoE(-/-) Mice Aorta and Human CEA Plaques

**Figure 1:**
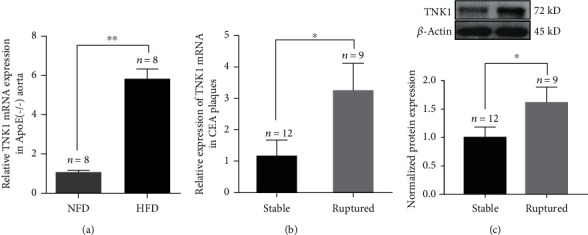
The TNK1 expression in ApoE(-/-) aorta and human CEA plaques. (a), the expression of TNK1 mRNA in NFD and HFD-fed ApoE(-/-) mice; (b), the TNK1 mRNA expression in human CEA plaques; (c), the TNK1 protein expression in human CEA plaques. All values are presented as mean ± SD. ^∗^*P* < 0.05; ^∗∗^*P* < 0.01.

After an 8-week treatment with HFD, obvious deposition of lipid was found in HFD-fed mice. The aorta arch of ApoE(-/-) mice were collected, and the TNK1 mRNA expression were detected. As shown in Figure 1(a), a significant increase in TNK1 mRNA was found in the aorta of HFD-fed mice [9]. 12 ruptured plaques and 9 stable plaques were collected from CEA. The characteristics and blood lipoprotein levels are shown in Table 2. In human CEA ruptured plaques, the levels of TNK1 mRNA and protein expression were also significantly increased (Figures 1(b) and 1(c)).

### 3.2. Expression of TNK1 in Different Cell Lines

**Figure 2:**
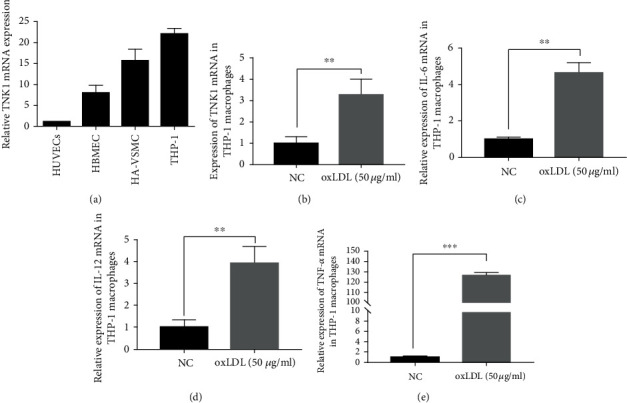
Effects of oxLDL on TNK1, IL-6, IL-12, and TNF-*α* mRNA expression in THP-1-derived macrophages. (a) The expression of TNK1 in different cell lines; (b-e) the effect of oxLDL on TNK1, IL-6, IL-12, and TNF-*α* expressions in THP-1 macrophages. All values were presented as mean ± SD from three independent experiments. ^∗∗^*P* < 0.01, ^∗∗∗^*P* < 0.001.

To explore the functions of TNK1 in atherosclerosis, we detected the expression of TNK1 in different cell lines, using GAPDH as reference. As shown in Figure 2(a), TNK1 was expressed in all of the HUVEC, HBMEC, HA-VSMC, and THP-1 cells. Among them, THP-1 cells were with the highest level of TNK1.

### 3.3. Effects of oxLDL on TNK1, IL-6, IL-12, and TNF-*α* mRNA Expression in THP-1-Derived Macrophages

Further experiments were conducted in THP-1 cells to investigate the functions of TNK1 on inflammation. 50 *μ*g/mL of oxLDL treatment significantly promoted the expression of TNK1, IL-6, IL-12, and TNF-*α* mRNA expression in THP-1-derived macrophages (Figures 2(b)-2(e)). The level of TNK1, IL-6, and IL-12 mRNA increased over 3-folds in the oxLDL group compared to the negative control (NC) group (Figures 2(b)-2(d)). The level of TNF-*α* mRNA increased more than 300-folds in the oxLDL group compared to the NC group (Figure 2(e)).

### 3.4. Effects of TNK1 on the Lipid Uptaken and Lipid Contents in THP-1-Derived Macrophages

**Figure 3:**
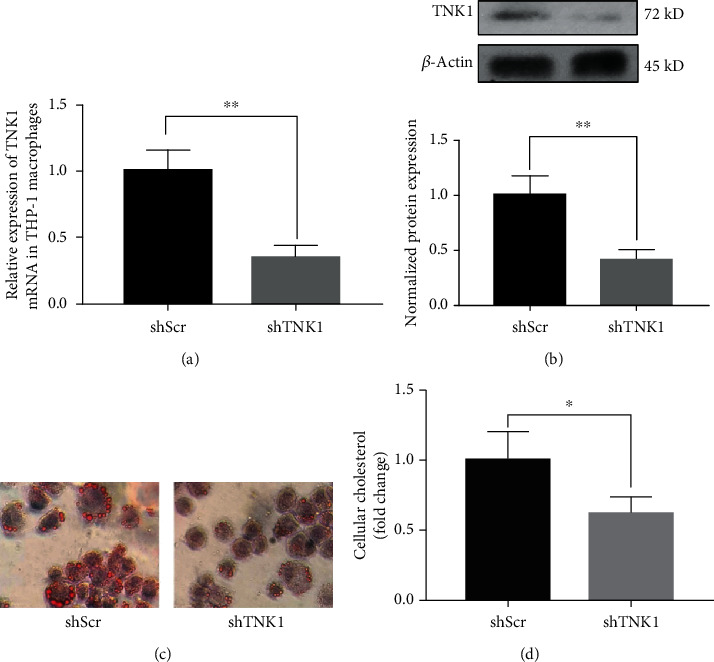
The effects of TNK1 on lipid uptaken and lipid content in THP-1-derived macrophages. (a, b) The suppression effect of shTNK1; (c) the uptake of oxLDL by THP-1 macrophages; (d) the intracellular total cholesterol in THP-1 macrophages. All values are presented as mean ± SD from three independent experiments. ^∗^*P* < 0.05; ^∗∗^*P* < 0.01.

As shown in Figures 3(a) and 3(b), shTNK1 inhibited the expression of TNK1 significantly. Inhibition of TNK1 decreased the amount of oxLDL uptaken by THP-1 macrophages (Figure 3(c)). Further measurement of intracellular total cholesterol indicated less content of cholesterol after suppression of TNK1 (Figure 3(d)).

### 3.5. Effects of TNK1 Downregulation on IL-12, IL-6, and TNF-*α* Expressions in oxLDL-Treated THP-1 Macrophages

**Figure 4:**
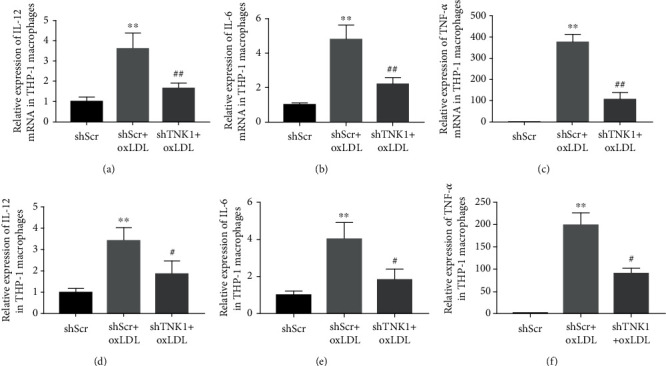
Effects of TNK1 inhibition on IL-12, IL-6, and TNF-*α* expressions. (a-c) The expression of IL-12, IL-6, and TNF-*α* mRNA expressions detected by qPCR; (d-f) the expression of IL-12, IL-6, and TNF-*α* protein expressions detected by ELISA. All values are presented as mean ± SD from three independent experiments. ^∗^*P* < 0.05; ^∗∗^*P* < 0.01, compared to shScr; ^#^*P* < 0.05, compared to shScr+oxLDL.

As shown in Figures 4(a)-4(c), oxLDL treatment increased the expressions of IL-12, IL-6, and TNF-*α* mRNA significantly. The inhibition of TNK1 suppressed the oxLDL-induced upregulation of these genes. The levels of IL-12, IL-6 and TNF-*α* in cell culture supernatant were also increased after oxLDL treatment and was suppressed through the inhibition of TNK1 by shTNK1 (Figures 4(d)-4(f)).

### 3.6. Effects of shTNK1 on oxLDL-Induced Tyk2 and STAT1 Phosphorylation in THP-1 Macrophages

**Figure 5:**
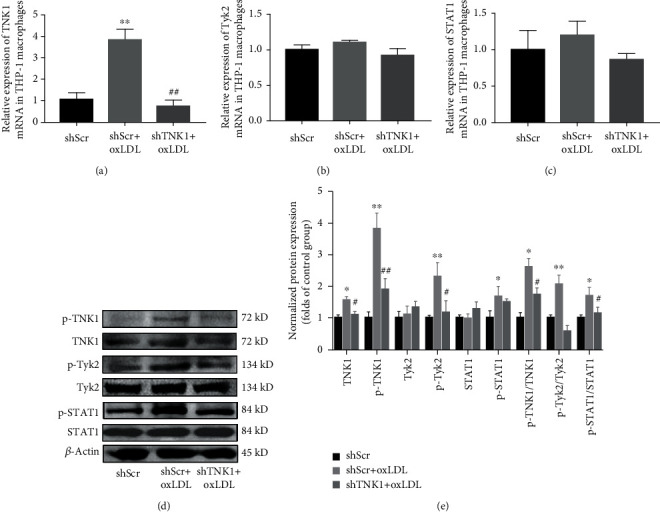
The effects of shTNK1 on oxLDL-induced alteration of different genes and proteins in THP-1 macrophages. (a-c) The relative expression of TNK1, Tyk2, and STAT1; (d) the protein expression detected by western blot; (e) relative quantified band density of different proteins. All values are presented as mean ± SD from three independent experiments. ^∗^*P* < 0.05; ^∗∗^*P* < 0.01, compared to shScr; ^#^*P* < 0.05, compared to shScr+oxLDL.

To further investigate the involvement of Tyk2/STAT signal in the functions of TNK1, the protein levels of TNK1, p-TNK1, STAT1, p-STAT1, Tyk2, and p-Tyk2 were measured by western blot. As shown in Figure 5(a), oxLDL promoted the expression of TNK1 mRNA significantly. The protein level of TNK1 also increased (Figures 5(d) and 5(e)). More importantly, oxLDL induced the phosphorylation of TNK1, Tyk2, and STAT1 significantly, with no influence on total expression of Tyk2 and STAT1. The ratio of p-TNK1/TNK1, p-Tyk2/Tyk2, and p-STAT1/STAT1 was all increased. The shTNK1 treatment inhibited the oxLDL-induced phosphorylation of TNK1, Tyk2, and STAT1 dramatically (Figures 5(d) and 5(e)).

## 4. Discussion

The present study found the upregulation of TNK1 in both HFD-fed ApoE(-/-) mice aorta and human ruptured plaques. The level of TNK1 is highly expessed in THP-1 cells, compared to other atherosclerotic related cells (HUVEC, HBMEC, and HA-VSMC), indicating TNK1 might be involved in the inflammation. Suppression of the expression of TNK1 inhibited the oxLDL-induced secretion of inflammatory factors and inhibited the uptake of lipid and decreased the cellular cholesterol content in THP-1 cells. The mechanisms might be related to the phosphorylation of Tyk2 and STAT1.

TNK1, a nonreceptor tyrosine kinase belongs to ACK family, was involved in virus-related immune responses [7]. Our studies demonstrated a significant increase of TNK1 in HFD ApoE(-/-) mice. This increase of TNK1 also existed in ruptured human CEA plaques. These results indicated that the TNK1 might play roles in the pathogenesis of atherosclerosis. We also found that shTNK1 inhibited the uptake of lipid in macrophages, which confirmed the roles of TNK1 in atherosclerosis.

Previous studies found TNK1 activated JAK/STAT1 signals [7]. STAT1 mediates the functions of several atherosclerotic stimuli, such as IFN-*γ*, TLRs, and IL-6 [13–15]. The activation of STAT1 results in augmenting of migration and proliferation of SMC, migration and adhesion of leukocytes in atherogenesis [16]. In intracellular antiviral innate immunity, TNK1 is a unique player [7]. In HCV infection, TNK1 is recruited from plasma and phosphorylated (activated). The activation of TNK1 leads to the phosphorylation of Tyk2. The p-Tyk2 subsequently phosphorylates STAT1 on the position of tyrosine 701 and serine 727 [7]. We compared the levels of TNK1 in 4 cell lines and found that THP-1 was highly expressed in human monocytes cell line THP-1. Because of the critical roles of monocytes and STAT1 in inflammation, we decided to investigate the functions of TNK1 in atherosclerotic inflammation. As a result, we found the phosphorylation of TNK1 promoted the activation of Tyk2 and STAT1. Activation of TNK1 also promoted the production of inflammatory factors. ShTNK1 inhibited the activation of Tyk2 and STAT1 and the production of inflammatory factors. These results indicated that TNK1 participated in the inflammation process of atherosclerosis. The mechanisms might be related to the activation of Tyk2/STAT signal.

Besides atherosclerosis, TNK1 also participates in several other diseases, such as atypical dementia, Alzheimer's Disease, and colorectal cancer [17–19]. In the trauma-induced intestinal injury and multiorgan failure, TNK1 induces cell apoptosis and the release of proinflammatory factors, IL-6 and TNF-*α*. The mechanisms were related to STAT phosphorylation and NF-*κ*B translocation [20]. These discoveries support our results on the functions of TNK1 in inflammation. STAT1 activation promotes M1 macrophage polarization, leading to the proinflammatory functions in tissues [21]. Whether TNK1 also participates in the macrophage differentiation or polarization still needs further study.

IL-12, IL-6, and TNF-*α* are proinflammatory factors produced by macrophages, lymphocytes, and smooth muscle cells in atherosclerotic plaques. In the atherosclerotic patients, these factors are increased significantly and they participate in almost all steps of atherogenesis [2, 22, 23]. OxLDL is a strong stimulator of IL-12, IL-6, and TNF-*α* secretion [24, 25]. Discovering the signals between oxLDL and proinflammatory factors might be an important direction to the prevention of the damages of oxLDL. We found a significant increase of TNK1 after oxLDL treatment in THP-1 cells, accompanied by increased productions of IL-12, IL-6, and TNF-*α*. ShTNK1 suppressed the effects of oxLDL. Therefore, TNK1 might be one of the mechanisms mediating the oxLDL-induced proinflammation. However, most of our experiments were conducted on THP-1 cells. The role of TNK1 in other macrophage cells is still uncertain.

In summary, TNK1 participated in the inflammation in atherosclerosis. shTNK1 suppressed the oxLDL-induced inflammation and lipid deposition in THP-1 cells. The mechanism might be related to the Tyk2/STAT signal.

## Figures and Tables

**Table 1:** The primers used for qPCR detection.

Gene name	Forward	Reverse
TNK1	GGACCAAGCGGAACCAGAACAAG	CTCCTCCTCCATGTGCCTACGG
Tyk2	GTGGCAGCAGTGGCAGGAAC	CTCAGCTCCAGGCACTTGTTGTC
STAT1	GGAACTTGATGGCCCTAAAGGA	ACAGAGCCCACTATCCGAGACA
IL-12	TAAGATGCGAGGCCAAGAATTA	TACTCATACTCCTTGTTGTCCC
IL-6	CCAGCTATGAACTCCTTCTC	GCTTGTTCCTCAC**TA**CTCTC
TNF-*α*	CGTGGAGCTGGCCGAGGAG	AGGAAGGAGAAGAGGCTGAGGAAC
*β*-Actin	TGACTGACTACCTCATGAAGAT	CATGATGGAGTTGAAGGTAGTT
GAPDH	GGTGTGAACCATGAGAAGTATGA	GAGTCCTTCCACGATACCAAAG
Tnk1-mice	GAAAACCCCCACACAATCAC	GCTCCACCTCCATAATCTTCC
*β*-Actin-mice	GACTGACTACCTCATGAAGAT	CATGATGGAGTTGAAGGTAGTT

**Table 2:** Characteristics of included patients.

Characteristics	No. of patients with ruptured plaques (*n* = 12)	No. of patients with stable plaques (*n* = 9)	*P* value
Age (years)	65.73 ± 11.27	55.25 ± 5.75	0.177
Male (%)	91.68 (11)	88.89 (8)	—
Body weight index	24.66 ± 3.28	22.69 ± 1.76	0.896
Smoking (%)	58.30 (7)	66.67 (6)	—
Alcohol (%)	50.00 (6)	55.56 (5)	—
TG	3.61 ± 1.20	3.59 ± 1.11	0.610
HDL	0.98 ± 0.17	0.82 ± 0.14	0.337
LDL	2.40 ± 0.33	1.84 ± 0.04	0.485
TC	1.35 ± 0.45	1.07 ± 0.31	0.446

TG: triglycerides; HDL: high density lipoprotein; LDL: low-density lipoprotein; TC: total cholesterol.

## Data Availability

All the data were presented in the article.
